# Structural equation modeling analysis of determinants of barriers to breast self-examination among Eastern Chinese women

**DOI:** 10.1371/journal.pone.0283525

**Published:** 2023-03-24

**Authors:** Jianwei Yu, Yizheng Gao, Hairuo Wang, Binghao Liu, Shunhua Zhang

**Affiliations:** 1 School of Medical Imaging, Bengbu Medical College, Bengbu, Anhui, PR China; 2 School of Clinical Medicine, Bengbu Medical College, Bengbu, Anhui, PR China; Mokpo National University, REPUBLIC OF KOREA

## Abstract

**Background:**

This study aimed to establish a structural equation model to determine the associations among knowledge of breast cancer, beliefs about breast self-examination (BSE), BSE practice, socio-economic status (SES), and barriers to BSE among Eastern Chinese women.

**Methods:**

An online cross-sectional correlational survey of 2026 women from Eastern China was undertaken by a self-administered questionnaire in 2020. Structural equation modeling was utilized for examining the interrelationships between BSE barriers and other variables.

**Results:**

Our results suggested that BSE barriers were significantly and negatively related to SES, BSE beliefs, and BSE practices (*β =* -0.176, *p* < 0.001 and *β =* -0.118, *p* < 0.001 and *β =* -0.435, *p* < 0.001, respectively). Among them, participants’ BSE practices had the strongest effects on BSE barriers, while the effect of breast cancer knowledge on BSE barriers was in an opposite direction (*β =* 0.177, *p <* 0.001).

**Conclusions:**

BSE barriers were influenced by SES, breast cancer knowledge, BSE beliefs and BSE practices. Our results warn that breast cancer prevention education should strengthen knowledge of practical methods rather than general knowledge. Therefore, intervention strategies designed to develop BSE and breast cancer prevention should take into account multiple factors, in particular finding more effective ways for the public to turn knowledge into a motivator rather than a barrier.

## Introduction

Breast cancer is the first most frequent malignancy and the number five cause of cancer deaths in women globally, with 2.3 million new cases and 685 000 deaths in 2020 [1]. More than half of the cases occurred in low-and middle-income countries (LMICs). China, as the largest LMIC country, also faces the heavy burden of breast cancer. In 2020, there were approximately 420,000 breast cancer cases among Chinese women, accounting for 19.9% of total new cases worldwide [1]. Moreover, breast cancer incidence in China has increased more than twice as fast as the global rates, especially in urban areas [2]. Breast cancer has become a major healthcare challenge for China, which poses a serious threat to the health of Chinese women [3].

Fortunately, earlier detection through screening can significantly alleviate the burden of this highly treatable disease and improve survival. Evidence has shown that 25%–30% of breast cancers can be controlled through early effective screening [4]. Recommended screening strategies include clinical breast examination (CBE) by a physician, mammography, and breast self-examination (BSE) [5]. Until now, mammographic screening has been deemed to be the best approach to identifying breast cancer early [6]. However, this approach is limited in LMIC settings because of its high cost and low availability.

BSE has a unique role in the initial diagnosis of breast cancer in the asymptomatic stage, compared with other screening techniques [7]. It is an easy, cost-effective, widely available method for detecting breast cancer, which does not require a hospital visit and specialized equipment [8]. In addition, BSE is also considered an important part of breast health education. It contributes to improving women’s breast health awareness, which empowers women to familiarize themselves with their breasts and detect breast abnormalities early. Harvey et al. [9] reported that more than 65% of breast lumps were detected by women themselves. Additionally, Newcomb et al. [10] found that women who performed BSE with proficiency had a decreased chance of dying from breast cancer. Consequently, BSE is of great importance in countries where breast cancer is on the rise, especially for LMIC’s limited health resources [7, 8]. Currently, there is no nationwide population-based screening program for breast cancer in China [11]. BSE may be considered an attainable strategy in empowering Chinese women to detect breast cancer early.

Despite the well-established benefits of BSE, many women are inactive and do not perform it regularly [12]. A study conducted in Nigeria indicated that only 5.3% of women performed BSE regularly [13]. Similar results were reported from studies in Yemen [14], and Ethiopia [15].

To implement effective promotion strategies, gaining a deeper understanding of barriers to BSE is of fundamental significance. Many studies have confirmed that lack of knowledge and weaker health beliefs are significant barriers to widespread BSE implementation encountered by women [16]. In addition, a study conducted in Nigeria reported that fear of detecting breast abnormality is an important factor affecting BSE uptake. The result of a study done among Malaysian women showed that not having enough privacy, embarrassment, unnecessary and spending too much time were also factors that influence BSE performance [17].

Low socioeconomic status (SES) is another significant barrier to BSE practice. SES is a potential determinant of health-protective behaviour, referring to an individual’s position in the social structure. It is generally assessed using income, educational attainment, and occupation. Previous studies demonstrated that women with higher SES had greater odds of performing BSE than those with lower SES [18], and more highly educated women tended to have higher BSE practice rates than those less educated [19]. In addition, a study by Akinyemiju et al. [20] found that higher SES women were more likely to be aware of the need for breast screening, with lower barriers.

Knowledge, Attitude and Practice (KAP) theory is a theoretical model used to study and promote human health behaviours [21]. KAP theory suggests that health knowledge is the basis for building positive attitudes and behaviour change, attitudes or beliefs are the motivation for behaviour change, and the formation of healthy behaviours is the final goal [21]. However, some studies from China and Singapore that have applied the KAP theory have found that health knowledge and attitudes are not always effective in influencing behaviour [22, 23], so we need to understand that there are limitations to the KAP theory and that it does not always fully explain changes in health behaviour. We need to consider more relevant factors. Based on KAP theory, we speculate that there is a link between barriers and other factors such as knowledge, beliefs, behaviours and SES. Therefore, we constructed a hypothetical model between breast cancer knowledge, beliefs of BSE, BSE practice, SES and barriers related to breast cancer among women in East China.

Nevertheless, the interrelationship between influencing factors and BSE barriers, and the mechanism of action, remains unclear and requires further research. In addition, knowledge, beliefs, and other factors are latent variables that are difficult to measure directly. The traditional statistical method is unable to elucidate the complex causal relationships when potential variables are concerned. Structural equation modeling (SEM) is a far superior multivariate analysis approach that compensates for the defects of the conventional statistical technique [24]. It is used to simultaneously estimate and quantify complex relationships between the observed and latent variables in a model framework [24].

This study aimed to explore the factors influencing barriers to BSE among Eastern Chinese women and the interrelationship between these factors using an SEM approach.

## Materials and methods

### Participants and data collection

We conducted this cross-sectional study in Anhui Province, Eastern China. The target population was women (single and married), older than 18 years. Women diagnosed with breast cancer were excluded. We adopted the convenience sampling method to issue an anonymous online questionnaire through WeChat (a Chinese messaging app) and e-mails in 2020. All submitted questionnaires were examined and verified by the submitter to ensure that they were completed in full. Finally, 2293 questionnaires were completed. After excluding invalid questionnaires, 2026 valid questionnaires were obtained, with an efficiency rate of 88.36%.

### Sample size calculation

We used a convenience sampling method to conduct this cross-sectional survey. Using the Kish Leslie formula [25], we determined the sample size required for this survey to be 423: *N = Z*^*2*^*P (1-P) d*^*2*^ (*Z* = 95% CI = 1.96, *P* = 27.5%, *d* = 0.05). Where the P-value is the BSE practice rate and is derived from previous studies on breast cancer screening behaviour among Chinese women [26]. In addition, the minimum required sample size was 508 after taking into account a 20% non-response rate. The total sample size used in this study was 2026, which exceeded the minimum requirement. The sample size was sufficient to ensure the credibility of the study results.

### Consent to participate and ethics approval

All participants volunteered to take part in the survey and were of legal age in China. All participants voluntarily clicked on the link to the online questionnaire and completed the questionnaire. Before completing the online questionnaire, all participants were informed of the purpose of the study, confidentiality of data and consent information, and provided verbal informed consent by clicking "Continue". This study was performed in line with the principles of the Declaration of Helsinki. Approval was granted by the Medical Ethics Committee of Bengbu Medical College (2018047).

### Hypotheses

Hypothesis 1 (H1): Breast cancer knowledge has a significant effect on barriers to BSE. Hypothesis 2 (H2): Beliefs of BSE have a significant effect on barriers to BSE.

Hypothesis 3 (H3): BSE practice has a significant effect on barriers to BSE.

Hypothesis 4 (H4): SES has a significant effect on barriers to BSE.

Hypothesis 5 (H5): SES has a significant effect on breast cancer knowledge.

Hypothesis 6 (H6): SES has a significant effect on beliefs of BSE.

Hypothesis 7 (H7): SES has a significant effect on BSE practice.

Hypothesis 8 (H8): Breast cancer knowledge has a significant effect on BSE practice.

Hypothesis 9 (H9): Beliefs of BSE have a significant effect on BSE practice.

Hypothesis 10 (H10): Breast cancer knowledge has a significant effect on beliefs of BSE.

**Fig 1:**
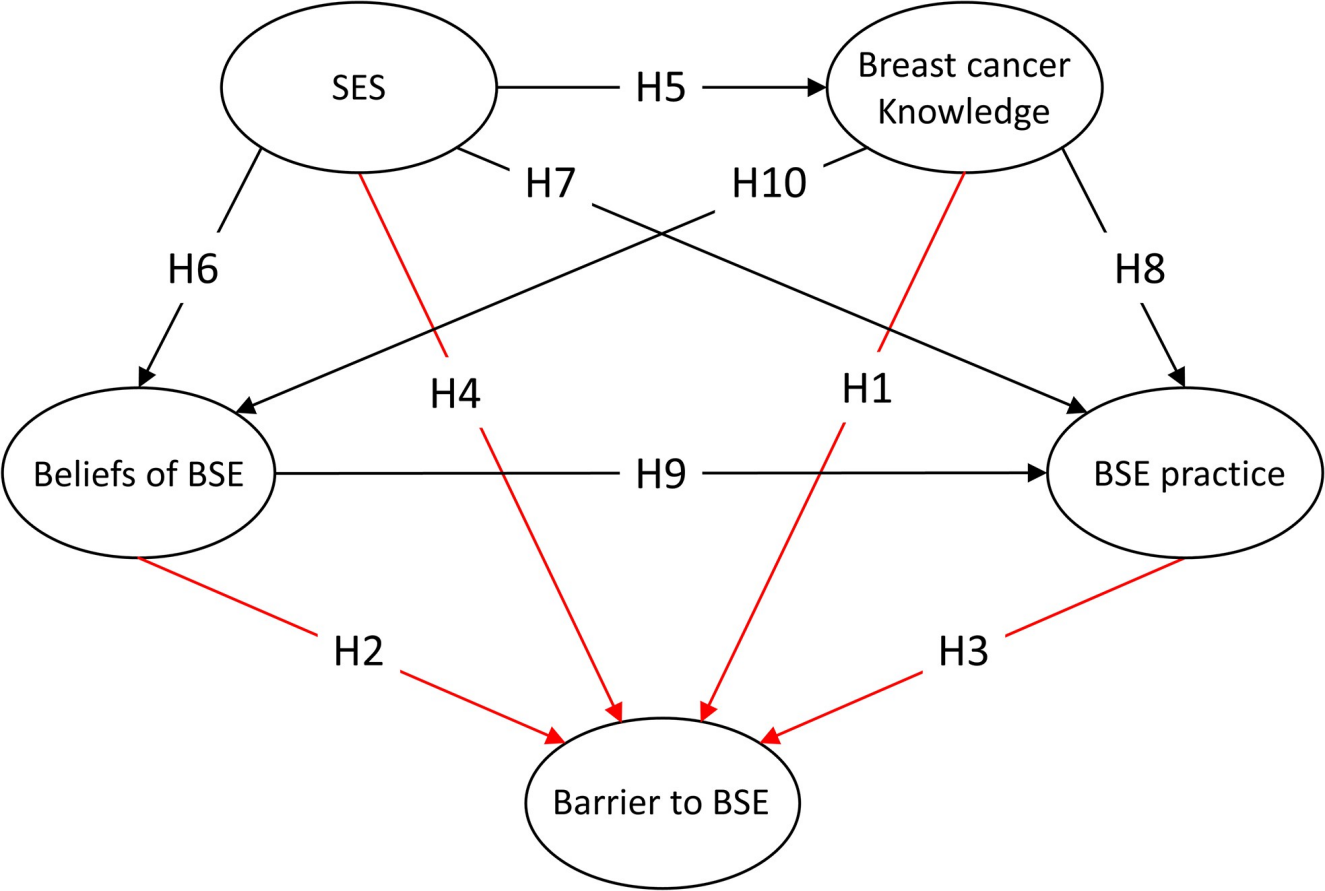
The original hypothetical model (M1). SES, Socioeconomic status; BSE, breast self-examination.

These hypotheses represent the relationships between potential variables in the form of a proposed structural model, as shown in Fig 1. The newly established connections (H1-4) in this study have been marked in red.

### Instruments

Most other previous questionnaires on breast cancer and BSE have focused on breast cancer knowledge, beliefs and BSE practice [27–30]. Questionnaires on SES and BSE barriers are rare. Based on an extensive review of previous studies [20, 31–33], A researcher-designed questionnaire was self-administered for the aims of our study and pre-tested on randomly sampled eligible participants before the formal survey. It addressed sociodemographic information, SES, breast cancer knowledge, beliefs of BSE, BSE practice and barriers to BSE. As the participants in this study were women from Eastern China, the language of the questionnaire used for the study and the language used for questionnaire administration was Chinese.

SES variables included current occupation (unemployed and retired/low-tech worker or farmer/intermediate technical worker or middle manager/senior technical worker or senior manager), education level (elementary school and below/junior high school/high school or secondary school / junior college/university/master/doctor), and annual household income (< 50,000 CNY (Chinese yuan renminbi)/50,000–120,000 CNY/ > 120,000 CNY).

Breast cancer knowledge was measured using 22 items, regarding risk factors, symptoms, screening methods and treatment strategies. Each item was rated as “true” (1 point) or “false” (0 points). Higher scores (range, 0–22) indicated better knowledge.

Seven items were used to assess the participants’ beliefs about BSE, for example, “It’s easy for me to keep up with regular breast exams or hospital visits”, “I believe I can handle unexpected situations during inspections” and so on. The responses were scored using a five-point scale ranging from 0 (strongly disagree) to 4 (strongly agree). High scores (range, 0–28) reflected a high-level belief of BSE.

The question “Do you have ever done BSE?” was first used to estimate BSE practice. Then those who had performed BSE were asked about the BSE frequency, techniques, and examination content. The responses to the BSE frequency were scored ranging from 1 (annually) to 4 (monthly). Response options for the other 3 questions were “No = 0” or “Yes = 1”. Higher scores (range, 1–7) represented better BSE practices.

Barriers to BSE were assessed using 5 items, for example, “I would be embarrassed to do a breast self-examination”. Each item had two options, which are “No” and “Yes” with scores of 0, and 1 respectively. Higher scores (range, 0–5) represented better BSE practices. A higher score of barriers to BSE (range, 0–5) indicated the significant likelihood of not participating in BSE implementation.

Cronbach’s alpha for this questionnaire was 0.814, KMO (Kaiser-Meyer-Olkin) was 0.825, and the *P*-value was 0.001, showing good reliability and validity.

### Data analysis

Data analysis was performed using statistical software IBM SPSS Statistics 26.0 and IBM SPSS Amos 24.0. The significance level was set at a *P*-value < 0.05.

Descriptive statistics were adopted to summarize the demographic information of the study sample. The overall mean of continuous variables (e.g., age) and the proportional distribution of categorical variables (e.g., ethnicity, marital status) were calculated to describe the whole independent sample. T-tests or analysis of variance were applied to compare the mean difference between the groups. Spearman’s correlation analysis was conducted to evaluate correlations between major variables. The SEM was employed to determine the relationships among study variables. Skewness and kurtosis tests were performed to assess the normality of observed data. The maximum likelihood estimation method was applied for parameter estimation.

### Indicators of model fit

A variety of goodness-of-fit indicators were applied in this study to evaluate the model fit of the hypothetical model. We used the chi-square value to degrees of freedom (*χ*^*2*^/*df*). *χ*^*2*^/*df* less than 5 is a sign of an overall acceptable model fit. We also used the comparative fit index (CFI) to evaluate the fit of the model. When the CFI is above 0.95, the model fit is good, with values below 0.9 indicating a poor fit. In addition, the goodness of fit index (GFI), Adjusted GFI (AGFI), adjusted goodness of fit index (RMSEA) and other fitness indices are also used to assess the model fitting.

### Modifications to the model

To get a better fit of the model, we used the modified indices method for the model modifications. The modified indices method is a way to improve the model by identifying parameters. The removal of insignificant paths (*p* > 0.05) and the addition of potentially significant ones are shown in the modification indices test. Nevertheless, our application of the modification indices was theory-based. It is important to avoid adding paths in a fully data-driven manner, as this is likely to lead to over-fitting.

## Results

### Characteristics of participants

The mean age of the 2,026 participants was 32.30 ± 12.204 years. Among them, 30.11% were over 40 years old and 47.38% were married. 17.08% of the participants had a history of breast disease. Over two-thirds of the participants had a university degree or higher. 45.31% of participants had annual household incomes between 50,000 CNY and RMB 120,000. 31.29% of participants had annual household incomes over 120,000 CNY. Univariate analyses indicated that breast cancer knowledge, beliefs of BSE, BSE practice and barriers to BSE were associated with most sociodemographic variables. Other demographic data are presented in Table 1.

**Table 1:** Participants’ demographic characteristics (n = 2026).

Variables	Total (%)	Scores (Mean ± SD)			
		Breast cancer knowledge	Beliefs of BSE	BSE practice	Barriers to BSE
**Age (years)**					
< 40	1416 (69.89)	16.80 ± 4.663	21.95 ± 3.394	2.82 ± 1.715	0.95 ± 0.961
> 40	610 (30.11)	16.17 ± 5.040	22.31 ± 3.753	3.81 ± 1.744	0.79 ± 0.920
*t/F*		9.496	-2.095	-11.808	3.573
*p-value*		0.002**	0.036*	< 0.001***	< 0.001***
**Birthplace**					
Urban areas	1183 (58.39)	16.60 ± 4.792	22.35 ± 3.603	3.38 ± 1.808	0.80 ± 0.891
Rural areas	843 (41.61)	16.63 ± 4.782	21.65 ± 3.332	2.75 ± 1.679	1.05 ± 1.012
*t/F*		0.04	4.421	5.842	4.173
*p-value*		0.841	< 0.001***	0.016*	0.041*
**Marital status**					
Single	1066 (52.62)	16.81 ± 4.604	21.83 ± 3.233	2.62 ± 1.658	1.02 ± 0.990
Married	960 (47.38)	16.38 ± 4.976	22.31 ± 3.778	3.68 ± 1.749	0.77 ± 0.889
*t/F*		7.838	-3.078	-13.992	5.996
*p-value*		0.005**	0.002**	< 0.001***	< 0.001***
**History of breast disease**					
Yes	346 (17.08)	16.53 ± 4.843	22.27 ± 3.230	3.58 ± 1.725	0.67 ± 0.755
No	1680 (82.92)	16.62 ± 4.777	22.02 ± 3.563	3.02 ± 1.779	0.95 ± 0.980
*t/F*		0.203	1.728	-5.349	5.090
*p-*value		0.652	0.189	< 0.001***	< 0.001***
**Occupation**					
Unemployed /retired	164 (8.09)	13.23 ± 5.197	21.38 ± 4.040	3.31 ± 1.785	0.79 ± 0.956
Low-tech worker / farmer	818 (40.38)	16.52 ± 4.656	21.80 ± 3.260	2.38 ± 1.561	1.07 ± 0.989
Intermediate technical worker/ middle manager	392 (19.35)	15.18 ± 4.844	21.91 ± 3.754	3.04 ± 1.665	0.83 ± 0.927
Senior technical worker/ senior manager	652 (32.18)	18.42 ± 3.977	22.65 ± 3.441	4.04 ± 1.672	0.76 ± 0.883
*t/F*		16.240	3.755	4.048	0.159
*p-*value		< 0.001***	0.011*	0.007**	0.924
**Education**					
Elementary school and below	19 (0.94)	12.79 ± 6.630	20.21 ± 5.391	2.53 ± 1.712	1.53 ± 1.467
junior high school	70 (3.46)	12.71 ± 5.582	21.04 ± 3.991	3.06 ± 1.744	0.80 ± 0.942
High school or secondary school	163 (8.05)	14.13 ± 5.220	21.83 ± 3.856	3.31 ± 1.823	0.97 ± 1.062
Junior College	372 (18.36)	16.48 ± 4.544	22.21 ± 3.832	3.62 ± 1.687	0.76 ± 0.933
University	1225 (60.46)	17.10 ± 4.511	22.09 ± 3.275	2.89 ± 1.770	0.96 ± 0.933
Master	172 (8.49)	17.87 ± 4.386	22.34 ± 3.537	3.55 ± 1.758	0.69 ± 0.881
Doctor	5 (0.25)	13.20 ± 7.497	22.60 ± 2.074	3.00 ± 1.581	0.80 ± 0.447
*t/F*		5.091	2.571	1.148	2.190
*p-*value		< 0.001***	0.017*	0.332	0.041*
**Annual household income**					
< 50,000 CNY	474 (23.40)	15.86 ± 5.148	21.32 ± 3.873	2.74 ± 1.689	1.01 ± 0.985
50,000–120,000 CNY	918 (45.31)	16.64 ± 4.636	22.27 ± 3.360	3.12 ± 1.741	0.92 ± .941
> 120,000 CNY	634 (31.29)	17.12 ± 4.656	22.31 ± 3.360	3.40 ± 1.856	0.79 ± .930
*t/F*		6.923	4.686	2.898	0.337
*p-*value		0.001**	0.009**	0.055	0.714

Note: CNY, Chinese yuan renminbi

*p < 0.05

**p < 0.01

***p < 0.001. BSE, Breast self-examination

### Correlation analysis among latent variables

Spearman’s correlation analysis indicated that barriers to BSE were negatively associated with SES (*r* = -0.116, *p* < 0.01), beliefs of BSE (*r* = -0.115, *p* < 0.01), BSE practice (*r* = -0.247, *p* < 0.01). The results are presented in Table 2.

**Table 2:** Mean, SD, and Spearman correlation coefficients among variables (n = 2026).

	Variables	M	SD	1	2	3	4	5
**1**	**Barriers to BSE**	0.90	0.951	-	-0.116**	0.010	-0.115**	-0.247**
**2**	**SES**	9.44	1.862		-	0.247**	0.142**	0.250**
**3**	**Breast cancer knowledge**	16.61	4.787			-	0.131**	0.185**
**4**	**Beliefs of BSE**	22.06	3.509				-	0.279**
**5**	**BSE practice**	3.12	1.782					1.00

Note

**p < 0.01. BSE, breast self-examination. SES, Socioeconomic status Supplementary

### Structural equation modeling analysis

**Fig 2:**
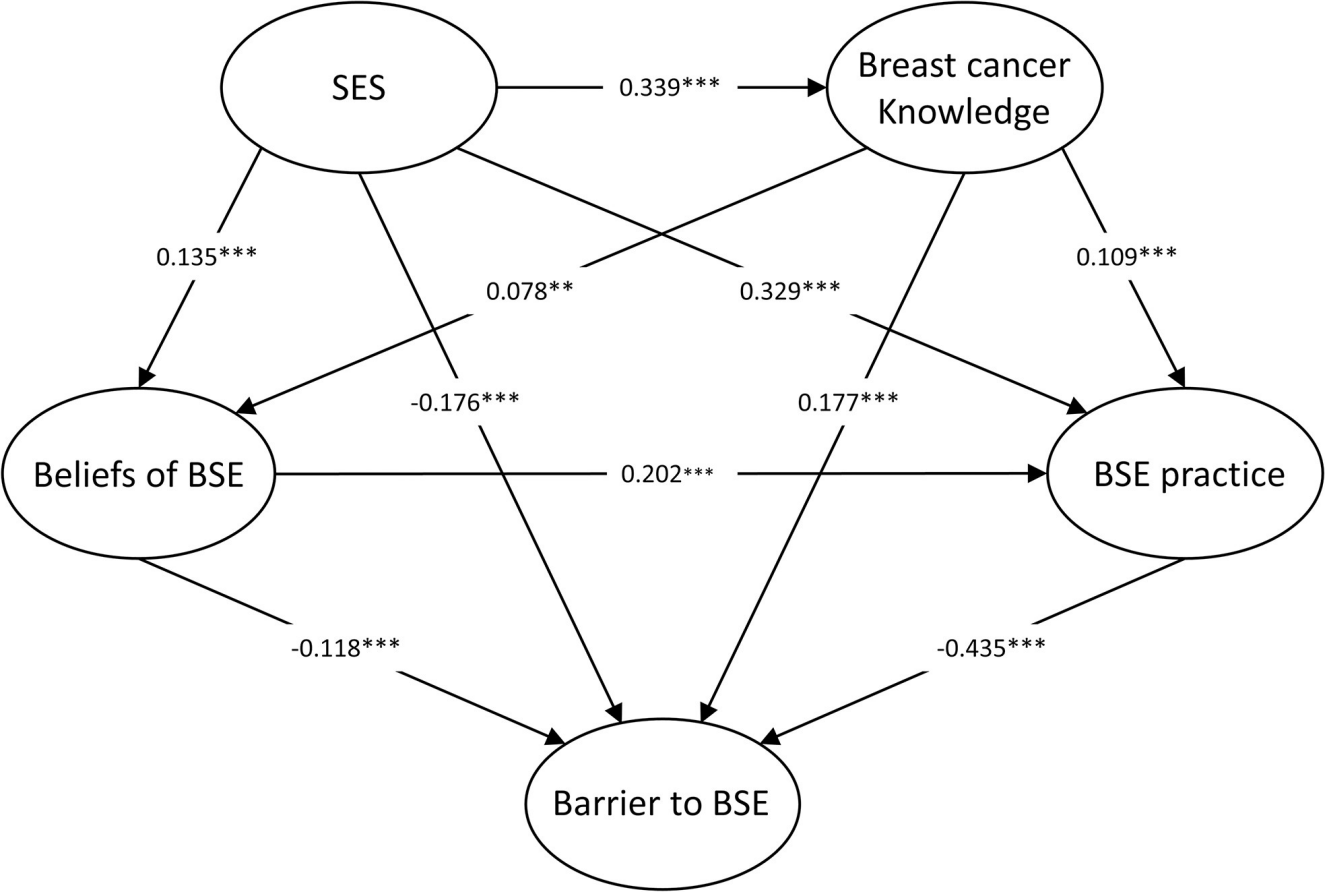
The final model (M2) with standardized path coefficients. ***p < 0.001; **p < 0.01; *p < 0.05. SES, Socioeconomic Status; BSE, breast self-examination.

As a result, all measured variables showed a normal distribution (Skewness < 3, Kurtosis < 8). The results of the SEM indicated the original hypothesized model (M1) did not fit the data well. According to modification indices and the theoretical background of AMOS, we concatenated some error terms of the same potential variables. After model modification, we obtained the final SEM model (M2) with the better fitting degree (Fig 2). Table 3 summarizes the fitness index of the SEM model M1 and model M2.

**Table 3:** Summary of model fitness index.

Fitness index	M1	M2	Recommended values
*χ* ^*2*^ */df*	25.304	4.510	≤ 5
GFI	0.844	0.958	> 0.90
AGFI	0.804	0.944	> 0.90
RMSEA	0.110	0.042	< 0.05
CFI	0.680	0.953	> 0.90
NFI	0.671	0.941	> 0.90
PGFI	0.672	0.712	> 0.50
IFI	0.680	0.954	> 0.90
PNFI	0.584	0.760	> 0.50

Note: AGFI, adjusted goodness of fit index; CFI, comparative fit index; *df*, degrees of freedom; GFI, goodness of fit index; IFI, incremental fit index; NFI, normed fit index; PGFI, parsimony goodness of fit index; PNFI, parsimonious normed fit index; RMSEA, root-mean square error of approximation; *χ*^*2*^, chi-square.

Moreover, the standardized path coefficients of the model M2 are statistically significant (all *p* < 0.01). The results of the path analysis indicated that participants’ barriers to BSE were directly and significantly negatively associated with SES (*β* = -0.176, *p* < 0.001), beliefs of BSE (*β* = -0.118, *p* < 0.001) and BSE practice (*β* = -0.435, *p* < 0.001), and was also positively related to breast cancer knowledge (*β* = 0.177, *p* < 0.001). Besides, BSE practice was directly correlated with SES (*β* = 0.329, *p* < 0.001), Breast cancer knowledge (*β* = 0.109, *p* < 0.001) and Beliefs of BSE (*β* = 0.202, *p* < 0.001). There was also a significant positive relationship between SES and Breast cancer knowledge (*β* = 0.339, *p* < 0.001). Tables 4 and 5 present the corresponding regression weights and standardized path coefficients of the model M2.

**Table 4:** Regression weight of M2.

Measured Variables	Estimate	S.E.	C.R.	*p*-value
SES → Barriers to BSE	-0.164	0.044	-3.716	0.000***
Breast cancer knowledge → Barriers to BSE	0.032	0.007	4.613	0.000***
Beliefs of BSE → Barriers to BSE	-0.056	0.016	-3.486	0.000***
BSE practice → Barriers to BSE	-0.228	0.023	-10.105	0.000***
SES → BSE practice	0.585	0.073	8.060	0.000***
Breast cancer knowledge → BSE practice	0.037	0.010	3.651	0.000***
Beliefs of BSE → BSE practice	0.184	0.023	7.961	0.000***
Breast cancer knowledge → Beliefs of BSE	0.029	0.011	2.646	0.008**
SES → Beliefs of BSE	0.264	0.067	3.967	0.000***
SES → Breast cancer knowledge	1.765	0.216	8.156	0.000***

Note

**p < 0.01

***p < 0.001. BSE, breast self-examination. SES, Socioeconomic status. S.E., the standard error. C.R., the critical ratio.

**Table 5:** Standardized regression weights of M2.

Measured Variables	Estimate	*p*-value
SES → Barriers to BSE	-0.176	0.000***
Breast cancer knowledge → Barriers to BSE	0.177	0.000***
Beliefs of BSE → Barriers to BSE	-0.118	0.000***
BSE practice → Barriers to BSE	-0.435	0.000***
SES → BSE practice	0.329	0.000***
Breast cancer knowledge → BSE practice	0.109	0.000***
Beliefs of BSE → BSE practice	0.202	0.000***
Breast cancer knowledge → Beliefs of BSE	0.078	0.000***
SES → Beliefs of BSE	0.135	0.008**
SES → Breast cancer knowledge	0.339	0.000***

Note

**p < 0.01

***p < 0.001. BSE, breast self-examination. SES, Socioeconomic status

## Discussion

This study investigated the relationship between breast cancer knowledge, BSE beliefs, BSE behaviours, SES and BSE barriers by constructing a structural equation model with a sample of women in Eastern China. The results revealed that breast cancer knowledge, BSE beliefs, BSE behaviours, and SES all had a significant effect on BSE barriers. BSE behaviours had the greatest effect on BSE barriers, compared to other factors. Moreover, BSE behaviours were strongly and significantly influenced by SES and BSE beliefs. Breast cancer knowledge and SES were significantly associated.

Here BSE practice was strongly associated with BSE barriers. Our research showed that participants with good BSE habits had lower barriers to BSE. BSE practice might increase women’s perceived motivation, positive beliefs and awareness of the benefits of BSE, as well as reduce the resistance and fear of BSE. They had a deeper understanding of the necessity of BSE and the dangers of breast cancer, and thus had lower barriers to BSE. In the study on BSE practice from University Tabuk in Saudi Arabia, similar views to ours were also mentioned [34]. A related study from Turkey also confirmed the role of BSE practices in enhancing women’s self-efficacy, increasing health motivation and reducing perceived barriers to mammography [35].

SES had a significant impact on BSE barriers and also a strong association with BSE practice and breast cancer knowledge. Our study showed that participants with higher SES had lower BSE barriers. One of the potential explanations for this phenomenon was that participants of higher social status, or with higher levels of education or higher income, were more aware of the dangers of breast cancer and more willing to undertake BSE for their health. From our results, we knew that civil servants, scientific researchers, medical workers or well-educated participants had a lower barrier to BSE and paid more attention to breast cancer. The research results of Madubogwu et al. [36] and Taleghani et al. [37] were also consistent with our findings. In addition, participants with higher household incomes spent more on healthcare and had greater access to health literacy training and medical services. This made them more knowledgeable about breast cancer and more open to BSE, lowering their barriers to BSE. It was clear from the results of the study that participants living in poor rural areas had higher barriers to BSE than those in urban areas. In a study from southwest Cameroon, Azemfac et al. reported that socio-economic disparities may be a determining factor in the spread of BSE knowledge and barriers to women’s care [38].

Another watchable result of our study was that breast cancer knowledge was significantly and positively linked to BSE barriers in participants. A study by Diyarbak from Turkey also found similar results to ours [39]. One of the underlying causes of this phenomenon was probably that breast cancer knowledge was not sufficient to reduce participants’ barriers to BSE in the absence of BSE practice. Our results indicated that only a small percentage of participants had regular BSE (17.7%). Perhaps this poor practice situation influenced the effect of breast cancer knowledge on participants’ BSE barriers. In addition, we found that the participants lacked knowledge of BSE, with 52.3% of the participants not knowing the correct examination methods. The lack of specific guidance information to motivate participants may be one of the reasons for this phenomenon. A study by X-L Gao et al. suggested that specific professional health knowledge had a greater impact on people’s health than general health knowledge [22].

Our model also suggested that positive BSE beliefs had a significantly negative impact on BSE barriers. Positive health beliefs can diminish participants’ fear, embarrassment resistance, and other negative psychology towards BSE. A survey of Malaysian women indicated that 61.5% of respondents had worries about BSE [17]. Many previous studies had indicated that fear, anxiety, and embarrassment were nonnegligible components of BSE barriers [6, 40]. This highlighted the importance of health belief factors in diminishing BSE barriers.

Our study is not flawless, and it still has some limitations as follows. The main limitation stemmed from our use of SEM, which made us cannot derive causality in the study. Notably, our participants are all from Anhui Province, China, which may cause our study to be non-nationally representative. In addition, there may be some flaws in the questionnaire design. In the more in-depth studies, we should conduct better longitudinal designs and expand sample sizes to address the above limitations.

## Conclusions

In conclusion, our study focused on the factors influencing BSE barriers among women in East China. BSE barriers were significantly linked with breast cancer knowledge, BSE belief, BSE practice, and SES. BSE practice had a stronger effect on BSE barriers. SES had a strong connection with breast cancer knowledge and BSE practice. Moreover, in general, good breast cancer knowledge can reduce BSE barriers. However, in our model, breast cancer knowledge had a stimulative effect on BSE barriers. These results suggested that prospective prevention of breast cancer should be more comprehensive and take into account multiple factors. In the dissemination of prevention knowledge, we may need to focus more on practical knowledge rather than general knowledge. It is imperative to find a more efficacious method for people to turn their knowledge into a motivator rather than a barrier.

## Supporting information

S1 File (XLSX)

S2 File (SAV)

S3 File (AMW)

## References

[1] Sung H, Ferlay J, Siegel RL, Laversanne M, Soerjomataram I, Jemal A, et al. Global Cancer Statistics 2020: GLOBOCAN Estimates of Incidence and Mortality Worldwide for 36 Cancers in 185 Countries. CA Cancer J Clin. 2021;71(3):209–49 doi: 10.3322/caac.21660 . 33538338

[2] Fan L, Zheng Y, Yu KD, Liu GY, Wu J, Lu JS, et al. Breast cancer in a transitional society over 18 years: trends and present status in Shanghai, China. Breast Cancer Res Treat. 2009;117(2):409–16 doi: 10.1007/s10549-008-0303-z . 19153831

[3] Chen W, Zheng R, Baade PD, Zhang S, Zeng H, Bray F, et al. Cancer statistics in China, 2015. CA Cancer J Clin. 2016;66(2):115–32 doi: 10.3322/caac.21338 . 26808342

[4] Azaiza F, Cohen M. Health beliefs and rates of breast cancer screening among Arab women. J Womens Health (Larchmt). 2006;15(5):520–30 doi: 10.1089/jwh.2006.15.520 . 16796479

[5] Leung J, McKenzie S, Martin J, Dobson A, McLaughlin D. Longitudinal patterns of breast cancer screening: mammography, clinical, and breast self-examinations in a rural and urban setting. Womens Health Issues. 2014;24(1):e139–46 doi: 10.1016/j.whi.2013.11.005 . 24439940

[6] Nde FP, Assob JC, Kwenti TE, Njunda AL, Tainenbe TR. Knowledge, attitude and practice of breast self-examination among female undergraduate students in the University of Buea. BMC Res Notes. 2015;8:43 doi: 10.1186/s13104-015-1004-4 . 25889644 PMC4414436

[7] da Costa Vieira RA, Biller G, Uemura G, Ruiz CA, Curado MP. Breast cancer screening in developing countries. Clinics (Sao Paulo). 2017;72(4):244–53 doi: 10.6061/clinics/2017(04)09 . 28492725 PMC5401614

[8] Dewi TK, Massar K, Ruiter RAC, Leonardi T. Determinants of breast self-examination practice among women in Surabaya, Indonesia: an application of the health belief model. BMC Public Health. 2019;19(1):1581 doi: 10.1186/s12889-019-7951-2 . 31775697 PMC6882356

[9] Harvey BJ, Miller AB, Baines CJ, Corey PN. Effect of breast self-examination techniques on the risk of death from breast cancer. CMAJ. 1997;157(9):1205–12 . 9361639 PMC1228347

[10] Newcomb PA, Weiss NS, Storer BE, Scholes D, Young BE, Voigt LF. Breast self-examination in relation to the occurrence of advanced breast cancer. J Natl Cancer Inst. 1991;83(4):260–5 doi: 10.1093/jnci/83.4.260 . 1994055

[11] Fan L, Strasser-Weippl K, Li JJ, St Louis J, Finkelstein DM, Yu KD, et al. Breast cancer in China. Lancet Oncol. 2014;15(7):e279–89 doi: 10.1016/S1470-2045(13)70567-9 . 24872111

[12] Ju N, Liao S, Zheng S, Hua T, Zhang S. Structural equation modeling to detect predictors of breast self-examination behavior: Implications for intervention planning. J Obstet Gynaecol Res. 2021;47(2):583–91 doi: 10.1111/jog.14550 . 33145891

[13] Amoran OE, Toyobo OO. Predictors of breast self-examination as cancer prevention practice among women of reproductive age-group in a rural town in Nigeria. Niger Med J. 2015;56(3):185–9 doi: 10.4103/0300-1652.160362 . 26229226 PMC4518334

[14] Al-Sakkaf KA, Basaleem HO. Breast Cancer Knowledge, Perception and Breast Self- Examination Practices among Yemeni Women: an Application of the Health Belief Model. Asian Pac J Cancer Prev. 2016;17(3):1463–7 doi: 10.7314/apjcp.2016.17.3.1463 . 27039790

[15] Abay M, Tuke G, Zewdie E, Abraha TH, Grum T, Brhane E. Breast self-examination practice and associated factors among women aged 20–70 years attending public health institutions of Adwa town, North Ethiopia. BMC Res Notes. 2018;11(1):622 doi: 10.1186/s13104-018-3731-9 . 30157951 PMC6114883

[16] Dagne AH, Ayele AD, Assefa EM. Assessment of breast self- examination practice and associated factors among female workers in Debre Tabor Town public health facilities, North West Ethiopia, 2018: Cross- sectional study. PLoS One. 2019;14(8):e0221356 doi: 10.1371/journal.pone.0221356 . 31437209 PMC6705765

[17] Akhtari-Zavare M, Juni MH, Ismail IZ, Said SM, Latiff LA. Barriers to breast self examination practice among Malaysian female students: a cross sectional study. Springerplus. 2015;4:692 doi: 10.1186/s40064-015-1491-8 . 26587360 PMC4642456

[18] Madan AK, Barden CB, Beech B, Fay K, Sintich M, Beech DJ. Socioeconomic Factors, not Ethnicity, Predict Breast Self-Examination. Breast J. 2000;6(4):263–6 doi: 10.1046/j.1524-4741.2000.99016.x . 11348376

[19] Yavari P, Pourhoseingholi MA. Socioeconomic factors association with knowledge and practice of breast self-examination among Iranian women. Asian Pac J Cancer Prev. 2007;8(4):618–22 . 18260740

[20] Akinyemiju T, Ogunsina K, Sakhuja S, Ogbhodo V, Braithwaite D. Life-course socioeconomic status and breast and cervical cancer screening: analysis of the WHO’s Study on Global Ageing and Adult Health (SAGE). BMJ Open. 2016;6(11):e012753 doi: 10.1136/bmjopen-2016-012753 . 27881528 PMC5129035

[21] Bettinghaus EP. Health promotion and the knowledge-attitude-behavior continuum. Prev Med. 1986;15(5):475–91 doi: 10.1016/0091-7435(86)90025-3 . 3774779

[22] Gao XL, Hsu CY, Xu YC, Loh T, Koh D, Hwarng HB. Behavioral pathways explaining oral health disparity in children. J Dent Res. 2010;89(9):985–90 doi: 10.1177/0022034510372896 . 20554887

[23] Qin Y, Zhang R, Yuan B, Xu T, Chen H, Yang Y, et al. Structural equation modelling for associated factors with dental caries among 3-5-year-old children: a cross-sectional study. BMC Oral Health. 2019;19(1):102 doi: 10.1186/s12903-019-0787-4 . 31170956 PMC6554934

[24] Kwok OM, Cheung MWL, Jak S, Ryu E, Wu JY. Editorial: Recent Advancements in Structural Equation Modeling (SEM): From Both Methodological and Application Perspectives. Front Psychol. 2018;9:1936 doi: 10.3389/fpsyg.2018.01936 . 30356842 PMC6190731

[25] Zheng S, Zhao L, Ju N, Hua T, Zhang S, Liao S. Relationship between oral health-related knowledge, attitudes, practice, self-rated oral health and oral health-related quality of life among Chinese college students: a structural equation modeling approach. BMC Oral Health. 2021;21(1):99 doi: 10.1186/s12903-021-01419-0 . 33676475 PMC7936478

[26] Bao Y, Kwok C, Lee CF. Breast cancer screening behaviors among Chinese women in Mainland China. Nurs Health Sci. 2018;20(4):445–51 doi: 10.1111/nhs.12533 . 29920900

[27] Bhandari D, Shibanuma A, Kiriya J, Hirachan S, Ong KIC, Jimba M. Factors associated with breast cancer screening intention in Kathmandu Valley, Nepal. PLoS One. 2021;16(1):e0245856 doi: 10.1371/journal.pone.0245856 . 33481894 PMC7822561

[28] Osei-Afriyie S, Addae AK, Oppong S, Amu H, Ampofo E, Osei E. Breast cancer awareness, risk factors and screening practices among future health professionals in Ghana: A cross-sectional study. PLoS One. 2021;16(6):e0253373 doi: 10.1371/journal.pone.0253373 . 34166407 PMC8224936

[29] Tuyen DQ, Dung TV, Dong HV, Kien TT, Huong TT. Breast Self-Examination: Knowledge and Practice Among Female Textile Workers in Vietnam. Cancer Control. 2019;26(1):1073274819862788 doi: 10.1177/1073274819862788 . 31304772 PMC6630076

[30] Hussain I, Majeed A, Masood I, Ashraf W, Imran I, Saeed H, et al. A national survey to assess breast cancer awareness among the female university students of Pakistan. PLoS One. 2022;17(1):e0262030 doi: 10.1371/journal.pone.0262030 . 35061770 PMC8782286

[31] Al Rifai R, Nakamura K. Differences in Breast and Cervical Cancer Screening Rates in Jordan among Women from Different Socioeconomic Strata: Analysis of the 2012 Population-Based Household Survey. Asian Pac J Cancer Prev. 2015;16(15):6697–704 doi: 10.7314/apjcp.2015.16.15.6697 . 26434897

[32] Nuche-Berenguer B, Sakellariou D. Socioeconomic determinants of cancer screening utilisation in Latin America: A systematic review. PLoS One. 2019;14(11):e0225667 doi: 10.1371/journal.pone.0225667 . 31765426 PMC6876872

[33] Donnelly TT, Al Khater AH, Al Kuwari MG, Al-Bader SB, Al-Meer N, Abdulmalik M, et al. Do socioeconomic factors influence breast cancer screening practices among Arab women in Qatar? BMJ Open. 2015;5(1):e005596 doi: 10.1136/bmjopen-2014-005596 . 25613951 PMC4305075

[34] Gonzales A, Alzaatreh M, Mari M, A AS, Alloubani A. Beliefs and Behavior of Saudi Women in the University of Tabuk Toward Breast Self Examination Practice. Asian Pac J Cancer Prev. 2018;19(1):121–6 doi: 10.22034/APJCP.2018.19.1.121 . 29373902 PMC5844605

[35] Avci IA, Kumcagiz H, Altinel B, Caloglu A. Turkish female academician self-esteem and health beliefs for breast cancer screening. Asian Pac J Cancer Prev. 2014;15(1):155–60 doi: 10.7314/apjcp.2014.15.1.155 . 24528018

[36] Madubogwu CI, Egwuonwu AO, Madubogwu NU, Njelita IA. Breast cancer screening practices amongst female tertiary health worker in Nnewi. J Cancer Res Ther. 2017;13(2):268–75 doi: 10.4103/0973-1482.188433 . 28643746

[37] Taleghani F, Kianpour M, Tabatabaiyan M. Barriers to Breast Self-examination among Iranian Women. Iran J Nurs Midwifery Res. 2019;24(2):108–12 doi: 10.4103/ijnmr.IJNMR_94_18 . 30820221 PMC6390439

[38] Azemfac K, Christie SA, Carvalho MM, Nana T, Fonje AN, Halle-Ekane G, et al. A Community-Based Assessment of Knowledge and Practice of Breast Self-Examination and Prevalence of Breast Disease in Southwest Cameroon. J Cancer Epidemiol. 2019;2019:2928901 doi: 10.1155/2019/2928901 . 30713554 PMC6333001

[39] Erdem O, Toktas I. Knowledge, Attitudes, and Behaviors about Breast Self-Examination and Mammography among Female Primary Healthcare Workers in Diyarbakir, Turkey. Biomed Res Int. 2016;2016:6490156 doi: 10.1155/2016/6490156 . 27123449 PMC4829675

[40] Akhtari-Zavare M, Lattif LA, Juni MH, Md Said S, Ismail IZ. Predictors affecting breast self-examination practice among undergraduate female students in Klang Valley, Malaysia. J Obstet Gynaecol Res. 2015;41(12):1982–7 doi: 10.1111/jog.12819 . 26554636

